# Evidence for the Role of Mitochondrial DNA Release in the Inflammatory Response in Neurological Disorders

**DOI:** 10.3390/ijms22137030

**Published:** 2021-06-29

**Authors:** Gonzalo E. Moya, Phillip D. Rivera, Kristin E. Dittenhafer-Reed

**Affiliations:** Department of Chemistry and Biology, Hope College, Holland, MI 49423, USA; gonzalo.moya@hope.edu

**Keywords:** mitochondrial DNA (mtDNA), mitochondria, inflammation, reactive oxygen species (ROS), neurodegenerative disease, neuropsychiatric disorder

## Abstract

Mitochondria are regarded as the metabolic centers of cells and are integral in many other cell processes, including the immune response. Each mitochondrion contains numerous copies of mitochondrial DNA (mtDNA), a small, circular, and bacterial-like DNA. In response to cellular damage or stress, mtDNA can be released from the mitochondrion and trigger immune and inflammatory responses. mtDNA release into the cytosol or bloodstream can occur as a response to hypoxia, sepsis, traumatic injury, excitatory cytotoxicity, or drastic mitochondrial membrane potential changes, some of which are hallmarks of neurodegenerative and mood disorders. Released mtDNA can mediate inflammatory responses observed in many neurological and mood disorders by driving the expression of inflammatory cytokines and the interferon response system. The current understanding of the role of mtDNA release in affective mood disorders and neurodegenerative diseases will be discussed.

## 1. Introduction

**Figure 1:**
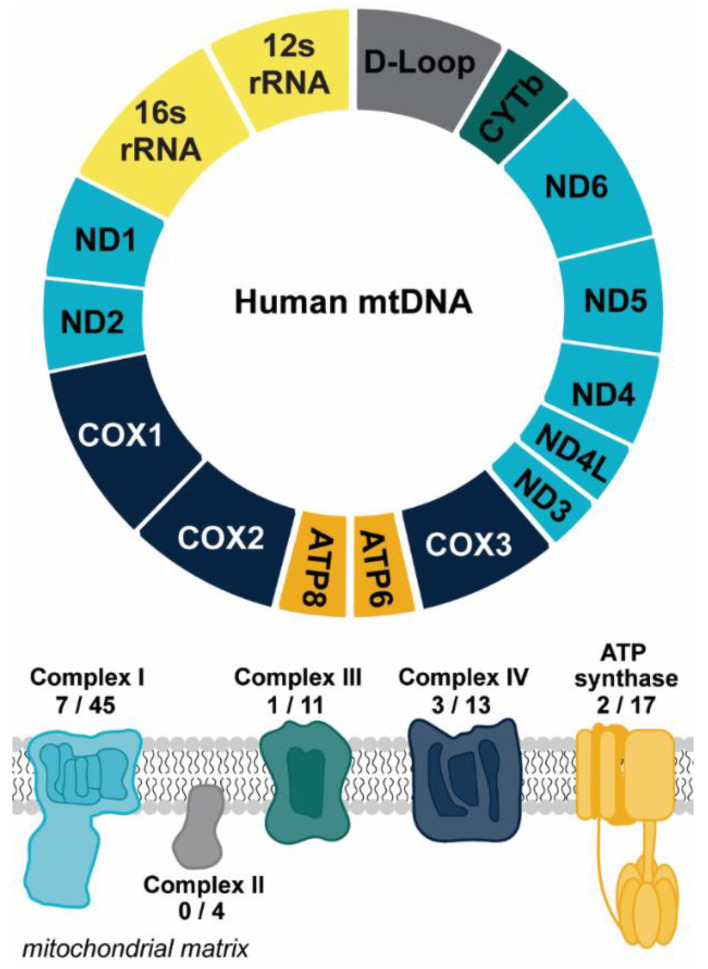
Overview of mtDNA. Mitochondrial DNA encodes for 13 protein subunits involved in oxidative phosphorylation (OXPHOS), the main energy-producing pathway of the cell. The diagram depicts the protein coding genes found within the mitochondrial genome that encode for subunits of OXPHOS complexes. Genes are color coded to correspond to the color of the OXPHOS complex. The numbers reflect the number of protein subunits of each complex encoded by the mitochondria out of the total number of protein subunits. The genetic instructions for the remaining subunits are carried in the nucleus. The 22 transfer RNAs encoded by the mitochondrial genome are not shown. Abbreviations: NADH dehydrogenase/Complex I subunits (ND1-6); Cytochrome oxidase/Complex III subunits (COX1-3); ATP synthase subunits (ATP6, ATP8); cytochrome b (CYTb).

Mitochondria are the metabolic hubs in eukaryotic cells. These organelles produce adenosine triphosphate (ATP) to fuel cellular functions and also play integral roles in multiple facets of metabolite processing (as reviewed in [1]). To maintain these functions, mitochondria rely on genetic information stored in the nucleus and their own small genome (mtDNA). Mammalian cells possess numerous copies of mtDNA, a 16.6 kilobase (kb) circular, double-stranded molecule encoding 13 proteins essential for electron transport and ATP synthesis, 22 transfer RNAs, and 2 ribosomal RNAs (Figure 1) [2]. The electron transport chain, apart from being vital for the generation of the proton gradient that drives ATP synthesis, is also the main generator of mitochondrial reactive oxygen species (mtROS). mtROS can act as an important redox signaling molecule, but exacerbated mtROS can lead to the damage of proteins, lipids, and nucleic acids that further drive mitochondrial dysfunction and apoptosis (as discussed in [1]). The increase in oxidative damage due to elevated mtROS is thought to contribute to the progression of many diseases [3]. One outcome of severe oxidative stress is the release of mtDNA from the mitochondria to the cytosol, which can be followed by extracellular release [4,5,6]. This extracellular release is of particular importance due to the implications of mtDNA mediating and/or contributing to inflammatory responses, driven by the bacterial-like nature of mtDNA [7]. In this review we will discuss the ways in which mitochondria contribute to pro-inflammatory signaling through mtDNA release, as well as the ways extra-mitochondrial mtDNA may contribute to the progression of neurological disease.

## 2. Mechanisms of Mitochondrial DNA Release

### 2.1. mtDNA Release in Response to Cellular Stress

Mitochondria play important roles in cellular signaling. Recent evidence shows that mitochondria and mtDNA are heavily involved in immunity and inflammation (as discussed in [8,9]). Accumulation of oxidative damage in the mitochondrion can impair respiratory function, eventually leading to oxidation of mtDNA and other mitochondrial components [10]. Due to the proximity to the formation of ROS in the mitochondrial matrix, mtDNA is susceptible to numerous forms of oxidative damage, including chemical modification to nucleotide bases and the sugar backbone, as well as double-stranded breaks [11]. Mitochondria possess repair mechanisms for these oxidative lesions, including many pathways akin to those found in the nuclear DNA damage response (as reviewed in [12]). When damage accumulates beyond the capacity of these repair systems, mitochondria can be selectively degraded by macroautophagy, the cellular recycling system [13,14]. The degradation of mitochondria can lead to the release of mtDNA into the cytosol where it may activate a broad range of innate cellular responses [15,16]. Through autophagy, virtually all cellular components can be degraded in the lysosome [17]. A structure defined as the autophagosome is formed, consisting of membrane components of the endoplasmic reticulum and several protein complexes. These proteins bind mitochondrial components, targeting specific mitochondria to be encapsulated within the autophagosome for degradation [18]. In this review, we refer to mitophagy as the selective turnover of mitochondria through the autophagic pathway.

An example of one of these essential protein complexes in mitophagy is the ubiquitin-carrier p62 [19]. Mitochondria are polyubiquitinated upon phosphorylation of p62, following PTEN-induced kinase 1 (PINK1) non-canonical import into mitochondria [20]. PINK1 phosphorylates ubiquitin (Ub), essentially labelling mitochondria for degradation. The PINK1-derived rise in poly-ubiquitin recruits the poly-ubiquitinase PARKIN, which amplifies the damage signal by tagging multiple mitochondrial membrane proteins (such as VDAC1/2/3 and Mfn1/2) with ubiquitin. PINK1 activity also leads to the recruitment of other protein factors involved in the mitochondrial trafficking process into the growing pre-autophagosome [21,22]. The formed autophagosome, tightly encapsulating a mitochondrion, will act as a carrier vesicle that will eventually fuse with the lysosomal membrane. The components of the autophagosome are discharged into the lysosome, at which point it is referred to as the autolysosome.

Among many autophagocytic enzymes, it has been reported that the DNase II enzyme degrades oxidized mtDNA in autolysosomes [23]. In cases where mtROS levels are exacerbated, the nuclease activity of DNase II is saturated, leading to some oxidized mtDNA fragments escaping mitophagy and being released into circulation in mammals. Upon entering circulation, these mtDNA molecules may interact with immune cells (e.g., neutrophils, macrophages) and activate an immune response [15,24]. It is not exactly known how oxidized mtDNAs avoid mitophagy and subsequently activate the immune system; however, it is speculated that mtDNA may accumulate in the cytosol of the degrading cell, which may amplify the immune response by secreting pro-inflammatory markers that recruit immune cells (as discussed in [25]). Additional work is necessary to explore if altered mitophagy promotes mtDNA escape by nonspecific mechanisms or if there is a specific molecular mechanism that promotes mtDNA translocation to an extracellular space.

An additional mechanism of mtDNA release from mitochondria is a result of increased permeability of the inner mitochondrial membrane. This is thought to occur when the mitochondrial permeability transition pore (mPTP) opens in response to increased Ca^2+^ concentration within the mitochondrial matrix as a consequence of oxidative stress [26]. Transient opening of the mPTP is a homeostatic mechanism to regulate levels of ions (i.e., H^+^, Ca^2+^), small molecules (i.e., H_2_O), metabolites (i.e., NAD^+^), and mitochondrial matrix proteins. However, long-lasting mPTP opening has been linked to loss of mitochondrial membrane potential, loss of ATP synthesis, mitochondrial swelling, and apoptosis. Even though the structure of the mPTP is not well characterized, it is thought that the mPTP is composed of the outer mitochondrial membrane, a voltage-dependent anion channel (VDAC), and adenine nucleotide translocase (ANT). It is speculated that cyclophilin D (CyD) and phosphate carriers are regulatory proteins of the mPTP [27]. More recent work has speculated that (F)-ATP synthase serves as the structure of the pore and associates with CyD [28]. The estimated diameter of the mPTP is about 1.4 nm, large enough for solutes and ions no greater than 1.5 kDa to diffuse through. The pore would also allow for small single-stranded (ss)mtDNA or double-stranded (ds)mtDNA molecules to be released to the cytosol [29].

Studying the release of mtDNA fragments under long-lasting opening of mPTP demonstrated that after induction of oxidative stress only fragments smaller than 700 bp were released. These fragments consisted of complete or partial genes coding the OXPHOS protein complexes, cytochrome c oxidase I (MTCO1), NADH dehydrogenase III (MTND3), and ubiquinol:cytochrome c reductase complex cytochrome b subunit (MTCYB) [30]. This could explain why exacerbated mtROS-related stress leads to decreased metabolic activity of the cell and in some cases apoptosis [4]. It has been shown that the VDAC subunit plays an important role in nucleic acid transport in mammals, but not ANT. In contrast, in yeast both of these components were important for nucleic acid transport through the mitochondrial membrane [31,32]. It has also been suggested that VDAC facilitates mtDNA fragment migration to the cytosol through a direct interaction between its N-terminus and mtDNA [4]. This interaction between mtDNA and VDAC is thought to occur between a triad of positively charged amino acids of VDAC and the negatively charged backbone of mtDNA; however, precise sites of interaction remain unknown.

Lastly, one crucial pathway of mtDNA release from the cell is that in which the apoptotic cell expels mtDNA into the bloodstream or other extracellular fluids, such as cerebrospinal fluid (CSF) [33]. The specific mechanism by which this occurs remains poorly characterized. One possibility is that after mitophagy occurs, the cell can directly release mtDNA into the bloodstream. It is also possible that as the cell membrane is degraded through apoptosis, mtDNA can leak into the bloodstream, where it becomes cell-free, circulating mtDNA (ccf-mtDNA). ccf-mtDNA can promote an inflammatory response through activation of pro-inflammatory signaling pathways in the cell upon take up from the bloodstream through endocytosis [34].

### 2.2. mtDNA Release as a Pillar for Immune and Inflammatory Processes

mtDNA escape into the cytosol or discharge into the bloodstream is inherently tied to immune responses. ccf-mtDNA can act as an exogenous signal, promoting signaling pathways in macrophages or neutrophils that lead to an immune response. Cytosolic mtDNA or mtDNA fragments can also act as endogenous signals and are generated in response to mitochondrial stress and dysfunctional mitophagic clearance of damaged mitochondria. The cellular responses to endogenous and exogenous mtDNA overlap. To avoid confusion, when we discuss some of the diseases and disorders and how they relate to mtDNA release-dependent inflammation, we will make the distinction between activation of intracellular mechanisms caused by the mitochondrial release of mtDNA to the cytosol, and immune responses from cells that take up ccf-mtDNA from the bloodstream. Released mtDNA may reside in many conformations. From an evolutionary standpoint, pattern recognition receptors can trigger innate immune responses after detecting hypomethylated CpG motifs found in bacterial DNA and also in mtDNA (as discussed in [35]). The functional specificity of this mechanism presumably does not discriminate between integral and fragmented mtDNA in either a linear or circular structure. As such, this review posits that mtDNA released in any form, including native mtDNA or fragments of mtDNA, can result in the immune responses described below.

**Figure 2:**
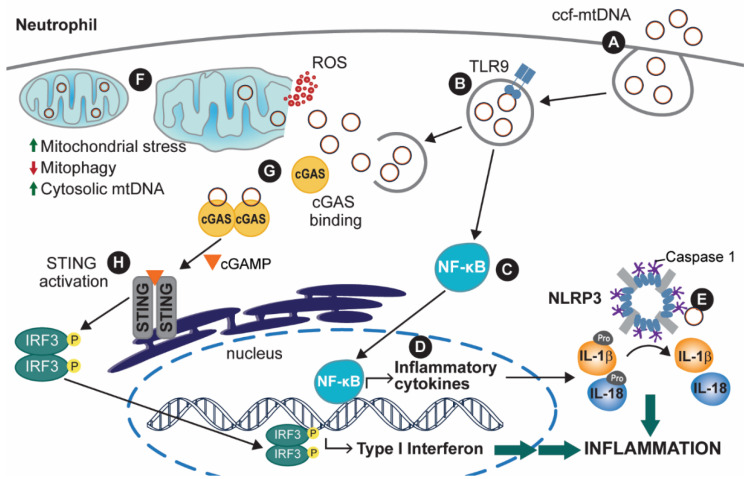
The inflammatory response after mtDNA release from mitochondria or ccf-mtDNA internalization. (**A**) Internalization of ccf-mtDNA leads to (**B**) activation of Toll-like receptor 9 (TLR9) and (**C**) upregulation of nuclear factor-κB (NF-κB), which (**D**) promotes the expression of inflammatory cytokines. Additionally, a rise in intracellular reactive oxygen species (ROS) causes (**E**) NOD-, LRR-, and pyrin domain-containing protein 3 (NLRP3) activation, ultimately leading to NLRP3 inflammasome assembly. This causes caspase-1 (CAS1)-mediated pro-interleukin (pro-IL) activation, leading to either localized inflammation or cytokine release. Alternatively, (**F**) mtDNA can be released to the cytosol and bind cyclic GMP-AMP synthase (cGAS) following endosomal rupture, or (**G**) mtDNA release from mitochondria. mtDNA release from the mitochondria can be a result of mitochondrial dysfunction or impaired mitophagy. (**H**) cGAS-bound mtDNA can lead to a type I interferon (IFN) response, which further contributes to the inflammatory response from (**E**). While mtDNA is depicted in an intact and circular form, mtDNA fragments in linear or circular form may also elicit similar immune responses.

When mtDNA is released into the bloodstream, it can be taken up by some immune cells, such as neutrophils, by endocytosis (Figure 2A). Upon compartmentalization of the engulfed mtDNA in an endosome (or endosome-like) vesicle, the endosomal Toll-like receptor 9 (TLR9) can be activated by mtDNA (Figure 2B). The activation of TLR9, a pillar of antibacterial and antiviral responses, is thought to occur because of the similarity between mtDNA and bacterial DNA, as both are rich in CpG-repeat regions [7,36]. Activation of TLR9 leads to subsequent activation of the transcription factor family nuclear factor kB (NF-κB), which is the leading cause of pro-interleukin (pro-IL) -1β and -18 expression [37,38] (Figure 2C,D). pro-IL proteins are inactive forms of the inflammatory IL cytokines, which are activated through post-translational editing of pro-ILs, more specifically, by being cleaved by caspases in the cell (Figure 2E) [39]. TLR9/NF-κB activation leads to expression of the tumor necrosis factor-ɑ (TNF-ɑ), which is associated with apoptotic responses to the accumulation of cellular stress and recruitment of other immune factors [40,41,42,43]. Lastly, NF-κB also plays a role in interferon (IFN) type I responses, commonly a cellular antiviral response [44,45]. In doing so, activated NF-κB factors are coupled with interferon regulatory factors (IRFs), which together lead to expression of IFN type I response-associated factors, thus activating cellular immunity and also upregulating TNF-ɑ expression [46].

Another inflammatory response that has been extensively characterized as a result of TLR9 activation is the activation of the NOD-like receptor, pyrin containing protein 3 (NLRP3) inflammasome (Figure 2E) [47]. Upon activation of the NLRP3 protein in the presence of oxidized cytoplasmic mtDNA, NLRP3 oligomerizes with other activated NLRP3 proteins, forming a multi-subunit protein complex with holoenzyme function [48]. Formation of the activated NLRP3 inflammasome involves the binding of multiple pro-caspase 1 (pro-CAS1) proteins, which by enzymatic action of the activated inflammasome, cleave pro-CAS1 into the activated caspase 1 (CAS1) protein [49]. In turn, CAS1 is crucial for inflammatory cytokine production. CAS1 stimulates the processing of pro-IL-1β and pro-IL-18 into their active cytokine forms, interleukins (IL) -1β and 18 [50].

The cGAS/STING pathway of interferon (IFN) type I response is also activated by cytoplasmic mtDNA. The IFN-mediated response is designed to counteract microbial infections and was shown to promote inflammation in various areas of the body and promote apoptosis through upregulation of TNF-ɑ [51,52,53,54]. The accumulation of extra-mitochondrial mtDNA is a result of either internalization of ccf-mtDNA through endocytosis (Figure 2A) or mtDNA that is endogenously released in response to a build-up of damaged mitochondria within a cell (Figure 2F), but has not yet been released extracellularly. In either case, the extra-mitochondrial mtDNA can bind to the cyclic GMP-AMP synthase (cGAS). This causes cGAS to dimerize with another mtDNA-bound cGAS protein (Figure 2G,H). Upon dimerization, the cGAS dimer is activated to form cyclic GMP-AMP (cGAMP). cGAMP acts as an endogenous signal activating the STING receptor located in the ER [55]. Activation of this receptor then stimulates the interferon regulatory factor 3 (IRF3) through phosphorylation and dimerization. Upon formation of the phosphorylated dimer, IRF3 translocates to the nucleus, where it serves as a transcription factor for IFN type I genes [56].

## 3. Analysis of a Potential Link between Mitochondrial Dysfunction and Progression of Neuronal Disease

Mitochondrial aberrancies may lead to mtDNA release and prompt an inflammatory response associated with multiple neurological diseases. mtDNA content and ccf-mtDNA in plasma and CSF have been analyzed in a number of these diseases (Table 1). With more research, it is possible that mtDNA levels could serve as a biomarker for these conditions. It is key to note that we hypothesize the inflammatory response driven by released mtDNA may provide a link between mitochondrial dysfunction and the progression of neurological disease. A good portion of the evidence discussed in this section has not been directly associated with mitochondrial mechanisms of mtDNA release, but we propose that some of the activated inflammatory responses are mtDNA dependent. Highlighting the importance of employing greater focus to study this phenomenon, we classified diseases in two broad categories, neurodegenerative diseases and neuropsychiatric disorders, to allow for the analysis of patterns of mitochondrial dysfunction within disease sub-groups.

### 3.1. Neurodegenerative Diseases

**Figure 3:**
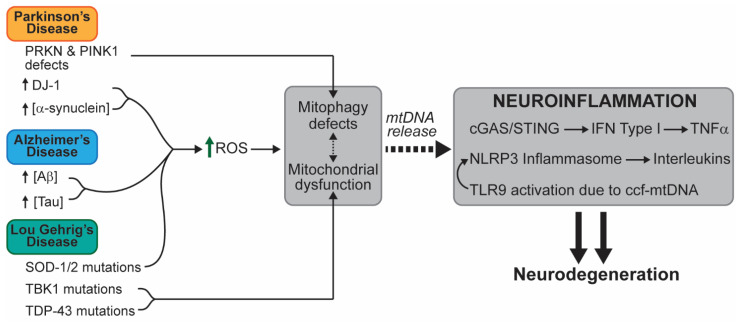
A schematic representation of mtDNA-mediated inflammation in relevant neurodegenerative diseases. The known alterations that lead to inflammation and neurodegenerative disease are presented. Different mutations or aberrancies lead to accumulation of damage in different ways. We propose that accumulation of defective mitochondrial due to impaired mitophagy and the accumulation of oxidative stress are common factors that link mtDNA-dependent inflammation within these pathologies. Abbreviations: amyloid-beta (Aβ); NOD-like receptor, pyrin containing protein 3 (NLRP3); reactive oxygen species (ROS); superoxide dismutase (SOD); TAR DNA-binding protein 43 (TDP-43); TANK-binding kinase 1 (TBK1).

The main etiologic characteristic of neurodegenerative diseases is the progressive loss of neuronal function leading to the devastating alteration of central nervous system (CNS) and peripheral nervous system (PNS) function. The symptoms of these diseases vary, ranging from motor loss to impaired cognitive function. We propose a link between the pathophysiology of neuronal degeneration and mitochondrial-associated patterns of inflammation (Figure 3).

#### 3.1.1. Amyotrophic Lateral Sclerosis (ALS)

##### mtDNA Release in ALS

Amyotrophic lateral sclerosis (ALS) is a disease characterized by progressive motor neuron loss leading to muscle weakness. While reports of ccf-mtDNA levels in ALS are inconclusive, multiple studies suggest a role for immune signaling initiated by endogenous mtDNA released into the cytoplasm, as well as abnormal or impaired mitophagic function in models of ALS. Some of these models are discussed below.

In healthy cells, the serine/threonine kinase TANK-binding kinase 1 (TBK1) and its downstream target optineurin (OPTN) are recruited to mitochondria following acute mitochondrial damage, coordinating engulfment by autophagosomes [77]. Mitochondrial recruitment to autophagosomes by TBK1 phosphorylation of OPTN is impaired in ALS, thus causing accumulation of damaged mitochondria and impairing mitophagy [78]. An in vivo model of ALS demonstrated increased mislocalization of the TAR DNA-binding protein (TDP-43) to the mitochondria when glutamine 331 was mutated to lysine [29]. An TDP-43 is a protein with established roles in ALS etiology [79]. The mitochondrial localization caused mtDNA release from the mPTP, as well as cytosolic accumulation of mtDNA, upregulating the activity of the cGAS/STING pathway and promoting an inflammatory response [29]. Apart from this report, Sieverding et al. [80] showed that the G298S mutation of TDP-43 increased TDP-43^G298S^ aggregation in the cytosol, impairing mitophagy. It has been shown that the p.G175S variant of TBK1 not only impaired assembly of the autophagosome, but also impaired degradation of TDP-43 [81].

As a reminder, in non-ALS reports, the impairment of mitophagy leads to the accumulation of mitochondria that build up oxidative stress, leading to mtDNA release [23,82,83]. In fact, some propose that mtDNA, along with other mitochondrial components, may be released into the cytosolic space following mitochondrial fragmentation and increased membrane permeability, as a result of exacerbated ROS production [84]. In their study, imbalances in glutamate signaling led to an increase in ROS production in addition to an increase in mitochondrial fragmentation and of cytochrome c and Opa1 release. Taking this into account, it would be interesting to explore the connection between mitochondrial fragmentation and the activation of CpG-mediated immune responses, which, if associated, could indicate a potential mechanism of mtDNA release as dependent on mitochondrial fragmentation.

##### Aberrant Mitochondrial Function in ALS

Several severe alterations in mitochondrial function and morphology have been reported in ALS. Perhaps the most characterized example of a mitochondrial aberrancy linked to ALS are SOD1 protein mutations [85]. SOD1 is a vital antioxidant that counteracts ROS in the cytosol [86]. In the absence of SOD1 function, cellular stress and macromolecular damage increases, and may lead to progression of disease [87]. SOD1 function is also tied to the activity of the mitochondrial Mn-dependent superoxide dismutase-2 (SOD2) [88,89], and in vitro and in vivo models of ALS showed reduction in SOD1 and SOD2 activity. Given that SOD2 is crucial in controlling mitochondrial oxidative stress, SOD2 impairment may lead to accumulation of mtROS. Collectively, SOD impairment creates a cellular environment prone to mitochondrial dysfunction, mtDNA oxidation, and mtDNA release into the cytoplasm.

Additionally, mutant forms of SOD1 in ALS aggregate preferentially on the outer mitochondrial membrane of spinal cord mitochondria [74,90,91,92]. This aggregation may impair the mitochondrial import machinery [93], leading to increased levels of glutamate in the synaptic cleft and increased expression of calcium permeable GluR2, resulting in an increase in postsynaptic intracellular calcium levels in motor neurons from ALS patients [92,94]. The abnormal rise in calcium signaling in ALS motor neurons may result in increased excitotoxicity in mitochondria due to calcium depletion in the endoplasmic reticulum and mitochondrial calcium overload [95]. Glutamate-derived excitotoxicity leads to drastic changes in mitochondrial membrane potential and contributes significantly to mitochondrial oxidative stress, and as such may be a potential mechanism by which neurons accumulate oxidative damage in ALS [96].

Another example of altered mitochondrial morphology and functions are polymorphisms of the fused in sarcoma (FUS) gene that have been reported to alter the shape and respiratory function of mitochondria in neuronal culture [97]. Mis-localized FUS interacts with the β-subunit of ATP synthase, which unfolds the protein [98]. It has also been shown that ALS-associated FUS interactions lead to dissociation of mitochondria with the endoplasmic reticulum, leading not only to considerable loss of cellular protein synthesis, but also to aberrant mitochondrial localization and shape [99]. In both cases, the altered function of the FUS protein disrupts normal mitochondrial morphology and bioenergetic function.

In relation to respiratory dysfunction of mitochondria, Vielhaber et al. [100] reported in a study analyzing myocytes from biopsies of ALS patients that about 65% of these had cell-wide defects in the NADH:CoQ oxidoreductase (Complex I) structure, explaining the loss of mitochondrial capacity to fuel muscle function, and thus leading to atrophy. As with many neurological disorders, there are a number of congenital DNA alterations that pose to be risk factors for ALS-onset (as discussed in [101]). A hereditary risk factor that is of particular importance is a polymorphism in the C9ORF72 gene in skeletal muscle cells of ALS patients that leads to increased TDP-43 expression [102,103]. Mutations to TDP-43 cause this protein to localize to the mitochondrion, binding mitochondrial mRNAs responsible for the expression of respiratory complex I subunits ND3 and ND6, thus impairing expression of these subunits and causing eventual complex I disassembly [104,105]. Not only do pathologic variants of the TDP-43 protein alter mitochondrial function, but so does increased activation of the VDAC1 and PHB2 mitophagic receptors [106,107]. Increased activation of these receptors causes intracellular TDP-43 levels to rise, the latter factor has been closely tied to mitophagy. Regardless of the rather incomplete characterization of the mechanism of action that gives rise to pathogenic TDP-43 function, it is important to note that whether due to direct genetic modifications of the protein itself, or due to intracellular imbalances, the main site of pathogenic action of TDP-43 is within the mitochondrion, severely impairing the OXPHOS machinery and mitochondrial function.

##### Inflammatory and Immune Responses in ALS

There have been several reports of abnormal mitophagic activity due to mitochondrial dysfunction or genetic aberrancies. In turn, we speculate that the altered mitophagic processes that may lead to abnormal mtDNA release may contribute in part to neuroinflammation in ALS.

There are many inflammatory processes proposed as being potential causes of ALS [108]. One example is the activation of TLR2 and TLR4 that is associated with increased IL-6 and IL-1β levels, which are also some of the main products of NLRP3 inflammasome-activation [109]. TLR2 and TLR4-activation can be a result of mtROS accumulation, which poses that the immune response in ALS may be mediated by both oxidative stress and mtDNA release [110]. It is also possible that with mtROS accumulation and impaired mitophagy comes mtDNA release, activating internal inflammatory mechanisms (such as the cGAS/STING pathway). Yu et al. [29] showed that mtDNA release resulting from mitochondrial internalization of TDP-43 led to an increase in cGAS/STING activation. If mtDNA is released out of the cell and becomes extracellular mtDNA, it can be taken up by nearby cells in the tissue and activate damage-associated molecular pattern (DAMP)-associated inflammatory mechanisms or activate immune cells. We believe that one way to determine if ccf-mtDNA is involved in the inflammatory pathogenesis of ALS is to measure the degree of TLR9 activation. Assuming no form of pathogen infection in the organism, a high degree of TLR9 activation in ALS (or ALS models) would signify that CpG containing mtDNA is activating an inflammatory response [111].

In ALS, there is not a substantial amount of research on the possible involvement of mtDNA release in the inflammatory and neurodegenerative etiology of the disease. However, considering the evidence on inflammatory molecular mechanisms in ALS and how these were previously linked to mtDNA release in other diseases, we consider that there is a significant amount of room for research to be done on this process. We speculate mtDNA plays a role in the neuroinflammatory response of ALS due to the presence of inflammatory markers (e.g., IL cytokines, TNF-ɑ) that correlate significantly with mtDNA release in other studies.

#### 3.1.2. Parkinson’s Disease (PD)

##### mtDNA Release in PD

Within the spectrum of neurodegenerative disorders that display motor symptoms, perhaps one of the most well characterized and studied disorders is Parkinson’s disease (PD). PD is characterized by progressive motor and non-motor neuron loss that causes tremors, impaired balance, and rigidity.

Over the past two decades, there has been increased interest in the involvement of mitochondria in PD, including numerous reports studying the extent of mitochondrial and mtDNA alterations associated with PD, particularly in substantia nigra neurons [112,113,114]. Multiple studies report that numerous mtDNA deletions cause alterations in expression and function of mitochondrial complexes I-IV of the ETC [115,116,117]. It is thought that one of the main alterations in mitochondrial function most associated with PD relates to mtDNA deletions affecting mitochondrial complex I activity (as reviewed in [118]). As such, there have been a number of reports that detail the increase in mtROS production in PD patients or models of PD [119,120,121]. It has been suggested that aberrations in PARKIN and PINK1-mediated mitophagy (a biomarker characteristic of PD) is what causes mitochondrial accumulation and mtDNA release in PD [122]. More specifically, upon loss of STING in Prkn^−^^/−^ and Pink1^−^^/−^ mouse models, the inflammatory response was attenuated. In a separate study, the CSF samples of PD patients with mutations in LRRK2, another protein crucial in regulating mitophagy, had significantly higher ccf-mtDNA levels than both idiopathic PD patients and healthy patients [123]. Treatment of PD patients with L-dopa significantly decreased CSF ccf-mtDNA levels compared to untreated PD patients [63].

Certain reports characterize conditions in PD neurons that could contribute to mtDNA release and report increases in ccf-mtDNA from non-idiopathic PD patients. However, there are studies reporting changes in ccf-mtDNA that appear to contradict each other (Table 1) [63,83,123,124]. It is important to note that these reports studied the changes in ccf-mtDNA in different gene mutation models of PD. As such, we are unable to propose a concrete link between PD neuroinflammation and mtDNA release, and thus further studies are critical.

##### Aberrant Mitochondrial Function in PD

Mitochondrial dysfunction has been reported as a major factor contributing to PD onset. Particularly, in early-onset PD, mitochondrial repair genes of the PRKN family (coding PARK proteins) and PINK1 are impaired, as reviewed in [125]. These genes encode proteins that are vital for effective mitochondrial clearing through mitophagy as described in the introduction. It is thus not surprising that reports of aberrancies to PINK1, PARK2, and PARK7 genes lead to abnormally high levels of mitophagy in substantia nigra neurons, further accelerating the neuroprogression of PD [126]. Borsche et al. [83] showed that mutations to PINK1 and PRKN genes lead to increased ccf-mtDNA levels. These findings support the general speculation that aberrant function of mitophagic proteins, specifically PINK1 and PARKIN proteins, may be one of the mechanisms for mtDNA release in PD. Additionally, it is important to note that carriers of PINK1 and PRKN mutations may be considered distinct from idiopathic cases of PD.

It has been proposed by some authors that the reason why there is an exacerbated amount of mitophagic proteins active in substantia nigra neurons is because of accumulation of oxidative stress from dysfunctional OXPHOS proteins. Specifically, it is thought that impaired complex I activity may be the factor giving rise to the motor symptoms of PD [127,128]. If mtROS-accumulating mitochondria do not have proper repair mechanisms to combat oxidative stress, then it would be plausible to tie the characteristic CNS atrophy of PD to aberrant mitochondrial function. With abnormal levels of mitochondrial degradation in substantia nigra neurons, one could attempt to explain why studies of PD-like neurodegeneration in cell cultures show significant decreases in mtDNA copy number, since loss of mitochondria will also lead to overall reduction of the respiratory capacity of substantia nigra neurons [112,129].

DJ-1 (also referred to as PARK7) is another protein that is important for the pathogenesis of PD as well as for other neurodegenerative disorders. DJ-1 is an antioxidant involved in the proteolytic pathway and is a biomarker for mitochondrial oxidative stress [130]. Mutations to DJ-1 have been linked to neurodegeneration and PD [131,132]. DJ-1 is recruited to the mitochondrion upon molecular sensing of oxidative stress [133] and scavenges mtROS [120]. In contrast, down-regulated expression of DJ-1 resulted in high amounts of cell death, which is thought to be due to the decrease in the activity of the potent antioxidant [121]. Several mutations of the DJ-1 gene that are associated with familial forms of PD caused significant alterations in mitochondrial bioenergetics [134,135,136,137]. In particular, Krebiehl et al. [136] reported that the E64D mutation of DJ-1 caused an increase in mtROS levels led to impaired mitophagy, and accumulation of dysfunctional mitochondria in a mouse model. Irrcher et al. [135] also reported significant increases in mtROS levels and Goldberg et al. [134] showed that DJ-1-knockout (KO) mice had decreased nigrostriatal dopamine levels, even though there was no significant evidence of neurodegeneration in the substantia nigra. This evidence suggests that a possible mechanism for mtROS accumulation in PD involves aberrations of DJ-1. Further work is required to determine if mtROS production through DJ-1 mutations could eventually lead to mtDNA release in PD.

Finally, increased mitophagic activity in PD may be due to mutations in Leu-rich repeat kinase 2 (LRRK2). There have been reports of an interaction between LRRK2 and PINK1, which renders particular importance to LRRK2 in the process of mitophagy [138]. Some of the mutations of LRRK2 associated with PD increase kinase activity [139]. Another common LRRK2 mutant associated with PD, G2019S, has been associated with oxidative damage of mtDNA due to impairment of mitophagy [140,141]. In PD-associated mutants of LRRK2, it has been reported that there are increased levels of mitochondrial fragmentation [121]. LRRK2 also coordinates microtubule-anchoring-protein degradation [142]. This may contribute to impaired mitochondrial autophagosomal translocation during mitophagy [143]. Finally, the G2019S mutant of LRRK2 has been associated with neurodegeneration in a mouse model of PD [144]. There are varied reports outlining the possible and distinct activities of LRRK2 in PD, warranting additional studies.

##### Inflammatory and Immune Responses in PD

As previously described, mtDNA release to the cytosol after exacerbated cellular stress is associated with increased NLRP3 inflammasome activity. Activation of the NLRP3 inflammasome leads to upregulation of inflammatory IL-1β and IL-18 [145]. In relation to PD, some PRKN and PINK1 KO mouse models of PD have displayed significantly high IL-6, IL-1β, TNF-α, and IFN-ɣ levels, alluding to exacerbated activation of the NLRP3 inflammasome and activation of the cGAS/STING pathway of IFN type I responses [122,146]. We speculate that impairment of mitophagy in PD may result in increased mtDNA release. This correlates with the increase in levels of activity of the ROS- and mtROS-dependent NLRP3 inflammasome and the corresponding IL inflammatory response from NLRP3/CAS1 activation and cGAS/STING-mediated upregulation of IFN genes. Mao et al. [147] demonstrated that in various neuroinflammation in vivo models of PD, substantia nigra of mice had increased levels of NLRP3 inflammasome expression, as well as IL-1β and caspase-1. Additionally, Wang et al. [148] showed substantia nigra microglia in PD exhibit exacerbated NLRP3 inflammasome activity due to increased mtDNA release after mtROS accumulation.

Inflammatory markers in PD are highly correlated with ROS-derived mtDNA release. Therefore, it is crucial to further investigate the extent to which mtDNA release, either from mitochondria or the cell, could be a plausible marker of neuroinflammation in PD.

#### 3.1.3. Alzheimer’s Disease

##### mtDNA Release in AD

Alzheimer’s Disease (AD) is characterized by the formation of amyloid-beta plaques in the extracellular space, as well as internal neurofibrillary tangles due to hyperphosphorylated microtubules [149]. Several brain structures suffer substantial neuronal loss with increased neuroprogression of AD, particularly in the medial-basal and medial- and lateral-temporal cortices [150]. The neuronal atrophy of AD, especially localized in the hippocampal area, is associated with a high degree of memory loss. Since the discovery of the disease in the beginning of the 20th century, several advances have been made in identifying some of the main markers associated with progression of the disease. These include the pathogenic form of β-amyloid (Aβ) that generates plaques in the extracellular space of the brain [151] and the intracellular accumulation of hyperphosphorylated Tau protein (p-Tau) that leads to the characteristic neurofibrillary tangles (NFTs) associated with neuronal loss [152].

A number of studies have noted an association between the rise in pathogenic Aβ levels and the impact of mtROS accumulation on mtDNA integrity [153,154]. It is thought that the increased oxidative damage in AD neurons is a product of the pre-symptomatic accumulation of Aβ plaques [155] and that Aβ could have a pro-oxidant effect in the neuron [156,157]. Intraneuronal accumulation of Aβ in non-AD adult brains occurs as a regular physiological mechanism that is a marker of organismal senescence [158]. In its pathological form, Aβ aggregates with redox-active metal ions (e.g., copper, zinc, or iron) which catalyze the production of extracellular ROS [159]. Microglia can clear these Aβ aggregates, but through this process, microglia accumulate a great degree of oxidative stress [160,161,162]. As previously discussed, accumulation of oxidative stress can severely impair mitochondrial function. In turn, mitochondrial dysfunction leads to an accumulation of mtROS. Specific to AD, exacerbated mitochondrial Ca^2+^ influx has been associated with mtROS accumulation in hyperglycemic amyloidogenesis [163]. Exacerbated mtROS levels in AD are associated with mtDNA damage [153,164] and the downregulation and dysfunction of OXPHOS enzymes, particularly complex IV [164,165,166].

One result of Aβ and p-Tau accumulation in the AD brain is mitophagy impairment [167,168,169,170]. Restoring mitophagy restored cognitive ability in mice [171]. Additionally, it is valuable to note that certain studies on AD patient biopsies or AD cell models revealed significant reductions in PARKIN and PINK1 [172,173], proteins crucial in the initial signaling process that triggers mitophagy. It was reported that PARKIN-deficient mice (not part of an AD model) showed increased cGAS/STING activation (and thus increased cytokine levels) and also found increased serum (ccf-) mtDNA [122]. Therefore, the missing link between mitophagy impairment and mtDNA-dependent inflammation in AD concerns the mechanism of release of mtDNA from damaged mitochondria into the cytosolic compartment or the extracellular space.

Overall, we speculate that accumulation of mtDNA damage may lead to a release of mtDNA to signal mitochondrial clearing and prevent cell death. However, there are reports of a significant decrease in CSF ccf-mtDNA levels in AD patients [62,64], but individual studies have failed to replicate these findings [25]. Others have reported that, apart from high degrees of variability in individual ccf-mtDNA levels, AD patients had significantly higher CSF mtDNA copies/µL compared to neurologically healthy controls [174]. The mixed evidence on changes in ccf-mtDNA levels in the AD brains highlight the importance of placing increased focus on mtDNA release as a potential biomarker of this neurodegenerative disease.

##### Aberrant Mitochondrial Function in AD

Many examples of mitochondrial dysfunction in AD are evident, including changes in mitochondrial morphology, number, and activity, collectively leading to the generation of mtROS. Cases of cognitive impairment or deficits were reported to be associated with downregulated expression of neuronal mitochondrial OXPHOS genes, possibly speaking to the metabolic imbalances of the disease that lead to severe neuronal loss [165,175,176].

Additionally, in the past decade, a genome-wide association study (GWAS) of late-onset AD has reported a particular polymorphism of a mitochondrial-acting nuclear gene, methylenetetrahydrofolate dehydrogenase (NADP^+^-dependent) 1-like protein (MTHFD1L), which plays a vital role in the reversible synthesis of 10-formyltetrahydrofolate into THF and formate in the mitochondrion [177,178]. This pathway is of vital importance in human health and is connected to homocysteine metabolism, DNA methylation, and DNA synthesis, as reviewed in [179]. Without proper MTHFD1L, homocysteine (HCy) clearing into methionine (Met) could be severely impaired [180,181]. HCy accumulation has been extensively associated with several forms of dementia [182]. Even though the link between HCy accumulation and the AD pathophysiology remains to be fully understood, it has been reported that impairment of folate metabolism, lower vitamin B_12_ levels, and accumulation of plasma HCy were associated with mild-cognitive impairment and AD [183]. Therefore, the relevance of the MTHFD1L polymorphism remains as a potential risk factor for late AD onset.

PET scan studies of early-onset AD patients indicated signs of brain hypometabolism [184]. This result by itself may seem inconclusive, since the etiology of numerous diseases includes brain hypometabolism, however in this study, this finding was correlated with cognitive decline. As several glycolytic and OXPHOS protein aberrancies in AD pathophysiology exist, cerebral hypometabolism may be eventually considered as a marker of AD progression. Up to now, the most definitive diagnosis of AD includes the identification of neurofibrillary tangles and amyloid plaques [185,186,187]. Therefore, brain hypometabolism may be a clear indicator of neurodegeneration and may be related to mitochondrial dysfunction that may exacerbate the rate of mtDNA release in the AD brain.

##### Inflammatory and Immune Responses in AD

Studies of the AD pathophysiological phenotype have reported upregulation of pro-inflammatory and pro-apoptotic factors in correlation with neurofibrillary tangles and Aβ plaques [188]. These inflammatory and necrotic factors include IL-1β, IL-6, IL-18, IFN-γ, and TNF-ɑ, as well as other upregulated pro-inflammatory genes such as NF-κB [189,190,191]. More specifically, and as we have discussed earlier, these factors are highly associated with activation of the NLRP3 inflammasome. Even though there is inconclusive evidence on whether ccf-mtDNA levels increase because of AD neurodegeneration, there are many pieces of evidence that allude to a mechanism of mtDNA release as a result of oxidative stress. As an example of this, Ahmed et al. [192] reported that NLRP3 inflammasome activation colocalizes with p-Tau and Aβ in glial cells of the AD brain. This report is of particular relevance given that it restates the characterized Aβ plaque clearing role of microglia and provides evidence that microglia may be involved in regulating mtDNA-related localized inflammation in the AD brain (as reviewed in [193]). It is known that activated microglia cause cytotoxic activation of astrocytes in AD pathophysiology, releasing inflammatory cytokines that lead to neuronal injury, and progressive loss of synapses [194,195,196]. More importantly, it has been reported that these cytokines are products of the activated NLRP3 inflammasome inflammatory response [191,197]. Glial NLRP3 inflammasome activation is associated with p-Tau upregulation and Aβ amyloid plaque formation due to microglial uptake and clearance of p-Tau and Aβ. The presence of inflammatory cytokines is also associated with NLRP3 inflammasome activation under severe states of oxidative stress (i.e., from exacerbated microglial Aβ uptake). Taken together, this could ultimately associate glial mtDNA release, as a product of oxidative damage from clearance of Aβ plaques in the AD brain, as a main contributor to local inflammation that leads to progressive neuronal loss [197].

Initially, Aβ is internalized by phagocytosis. The process of Aβ clearance causes significant oxidative damage to the cell, which has been shown to activate the NLRP3 inflammasome and cause ionic imbalances within the cell including, K^+^, Na^+^, and Ca^2+^ [198,199,200]. Specifically, P2X7 receptor-mediated imbalances in K^+^ and Ca^2+^ have been associated with severe oxidative stress accumulation in mitochondria [201,202]. Under these conditions, ROS production can promote mitophagy [203]. However, in certain cases, microglia are not able to clear dysfunctional mitochondria due to alterations in the genes encoding the primary mitophagic machinery (as reviewed in [204]). This is thought to exacerbate the inflammatory response through excessive release of mtDNA and cytokines. In non-AD cases, the extent of oxidative stress and inflammatory activation in the cell was used as a predictor for cell death [205,206], and thus we consider that it is important to evaluate whether this pattern is also found in AD cases with clear impairments of mitophagy.

A large body of evidence alludes a role for mtDNA release in promoting neuroinflammation and progression of disease. Therefore, we consider that it is critical that further research be performed exploring the phenomenon of mtDNA release in neurodegenerative diseases.

### 3.2. Neuropsychiatric Disorders

Neuropsychiatric disorders are characterized by alterations in brain function that lead to significant modifications in personality or behavior, having a great impact in the lives of those with the disorders and those of kin. Table 1 summarizes the results of a list of relevant studies on some diseases and disorders that share the common characteristic of localized neuroinflammation. Here, we focus on major depressive disorder as we believe it presents the most convincing case for the role of mtDNA and mitochondrial dysfunction in neuroinflammation. Other disorders that have received attention over the past years include schizophrenia (SZ) and bipolar disorder (BD). We have refrained from going in depth on these disorders due to the incongruent or insufficient reports on changes in ccf-mtDNA levels.

#### 3.2.1. Major Depressive Disorder (MDD)

##### mtDNA Release in MDD

Major depressive disorder (MDD), otherwise referred to as clinical depression, is an affective mood disorder characterized by anhedonia (lack of pleasure), sleep disturbances, and a lasting feeling of sadness or worthlessness. Generally, a disorder as such is not apparent to the clinician through clear-cut changes in brain morphology, but there is an evident change in cognitive function that alters mood and psyche.

It has been reported that ccf-mtDNA levels increase to a similar extent after psychosocial or physical stress [207]. Over the past decade, various studies reported an increase in ccf-mtDNA from samples (blood or CSF) obtained from MDD patients or animal models of MDD [68,69,208] (Table 1). However, the study of mtDNA copy number has not yielded as consistent of results [70,209]. The high degree of variability of mtDNA copy number from samples from MDD patients may represent the greater reliability of ccf-mtDNA measurements to monitor MDD progression.

##### Aberrant Mitochondrial Function in MDD

It has been proposed that MDD onset and progression are linked with changes in mitochondrial function, since drastic changes in metabolic function may be a leading cause for onset of the psychiatric symptoms. Even though it is subject to high variability, it has been reported that a decrease in mtDNA copy number correlates with MDD onset [210]. This may decrease metabolic function, leading to the characteristic changes in speed of cognitive activity or overall activity. There is also an association between mitochondrial complex I dysfunction and the onset of depressive symptoms, which supports this claim [211,212]. Certainly, onset of depressive symptoms, as it is with brain hypometabolism, is not a definitive diagnosis of clinical depression; however, this could be an explaining factor of decreased cognitive function in MDD due to impaired energy production from respiration [213,214,215].

Interestingly, in light of the previously discussed connection between MTHFD1L polymorphisms and AD, it has also been shown that a different polymorphism of this MTHFD1L (C665T) poses a high risk for MDD onset [216] and reduced PGC1α promoter methylation serves as a biomarker for MDD [217]. Downregulated expression or impaired function of MTHFD1L affects S-adenosylmethionine (SAM)-dependent methylation, which is the process by which CpG dinucleotides are methylated, such as those in the PGC1α promoter region. PGC1α induces the transcription of several metabolic and mitochondrial genes, providing a potential link between MTHFD1L defects, hypometabolism, and MDD.

Other genetic variants have either been directly associated or considered as risk factors for MDD, including an increase in hypothalamic-pituitary-adrenal (HPA)-axis activation [69,207,218]. This may be due to accumulation of oxidative stress in neurons [209,219], which as we have described previously, may lead to accumulation of damaged mtDNA and neuroprogression and inflammation in clinical depression (as discussed in [220]).

##### Inflammatory and Immune Responses in MDD

Increasing evidence indicates that a variety of inflammatory molecular markers associated with mtDNA release are present in high levels in diagnosed MDD patients. These molecular markers include IL-1β, IL-6, IL-8, IFN-γ, TNF-ɑ, and NF-κB [221,222]. Interestingly, some of the inflammatory cytokines mentioned (i.e., ILs) have been shown to decrease with antidepressant treatments (i.e., selective serotonin reuptake inhibitors), which seems to support the hypothesis that some type of cellular stress in the CNS first leads to inflammation, and second, expression of the depressive symptoms of MDD [223,224].

There are a wide variety of physiological mechanisms leading to a pro-inflammatory response, and therefore it is important to assess which seems to be the main underlying cause for cytokine-, pro-inflammatory-, and pro-apoptotic-factor expression. mtROS-dependent mtDNA release from a mitochondrion activates the NLRP3 protein receptor, forming a multi-subunit complex that leads to the subsequent activation of CAS1. The latter protein is then responsible for activating cytokines IL-1β, IL-8, and IL-18, and thus triggering an intracellular inflammatory response, or activating lymphocytes or neutrophils [50,225]. Recent reports have recorded high levels of inflammatory cytokines, high NLRP3 inflammasome, and increased CAS1 activity in murine models of MDD and in diagnosed MDD patients [226,227,228]. It is interesting to note that Alcocer-Gómez et al. [226] found that treatment with a number of different antidepressants reduced inflammatory cytokine levels and alleviated depressive behavior in a mouse MDD model and MDD patients. This study also found antidepressants hindered autophagy-dependent NLRP3-activation. Certainly, this study and the trend found in reports over the past decade allude to there being increased levels of localized inflammation in MDD patients.

In fact, the amount of evidence with regards to inflammatory markers in MDD has coined the inflammatory and neuroprogressive hypothesis of depression (as discussed in [220]). This hypothesis proposes that accumulation of cellular stress triggers cell-mediated-immune responses that seek to counteract tissue damage through immune system activation. In turn, this causes localized inflammation that may lead to a rise in the level of inflammatory cytokines [75]. It has been shown that an increase in cytokine levels in MDD correlates with HPA-axis activation, specifically through activation of NF-κB, IFN-γ, and IL-6 [229]. Collectively, it appears that some of the cellular mechanisms to counteract increased mtROS-related stress are highly prevalent in MDD and link abnormal HPA-axis activation with heightened localized inflammation in the MDD brain. Thus, we propose that mtDNA release may be one mechanism by which local inflammation occurs following cell-mediated immune activation.

An additional mechanism proposed in MDD involves activation of the TLR9 receptor. TLR9 expression in MDD patients is upregulated and expression levels can be normalized with antidepressant treatment [230]. This is important because TLR9 is activated by CpG DNA such as mtDNA. In macrophages and dendritic cells of MDD, TLR9 was upregulated in cells that were not infected with bacteria, suggesting that some other paracrine or tissue-specific factor may promote TLR9 upregulation following localized inflammation [231]. As stated, the fact that TLR9 is upregulated in MDD patients does not obligately mean that mtDNA causes such change; however, we believe that the degree of the local inflammatory response associated with NLRP3 inflammasome activation requires further investigation to determine if NLRP3 activation indeed occurs in a TLR9-ccf-mtDNA-dependent manner in MDD.

## 4. Conclusions and Outlook

We have discussed the presence of aberrancies in mitochondrial genes or mitochondrial enzymes that leads to progressive respiratory and metabolic impairment. In turn, this causes oxidative stress accumulation in the mitochondria. If oxidative stress is uncontrolled, it can lead to exacerbated damage to the mitochondrion and mtDNA can be released through mitophagy or cell apoptosis. mtDNA release triggers an inflammatory and immune response, thus contributing to nerve tissue loss that if undetected or untreated, can lead to the progression of any of the aforementioned maladies.

There is a great deal of synergy between mitochondrial dysfunction and the overall progression of neurodegenerative disorders. It is important to investigate whether mitochondria are also involved in neuroinflammatory processes of other neurological diseases that have yet to receive attention, such as addiction. The panorama for mitochondrial research in disease should not be restricted to alterations in metabolism or respiration. We propose that it would be interesting to deem mtDNA release-dependent inflammation in cases of disease as a potential link between metabolic or respiratory dysfunction, oxidative stress, and neuronal atrophy or neuroinflammation.

To conclude, ccf-mtDNA has the potential to be a reliable biomarker for neurological disease. However, to use these tools, it is critical to understand the link between mtDNA release, mitochondrial dysfunction, and disease. These details may allow for differential criteria of analysis or allow for one to predict disease onset. Therefore, to characterize the connection between mitochondria and neurological diseases, it is crucial to first analyze if inflammatory responses in a particular disease are associated with mtDNA release. If such connections are present, this may allow further investigation to understand how localized mitochondrial dysfunction leads to inflammation that is dependent upon released mtDNA.

## Figures and Tables

**Table 1:** A summary of studies measuring ccf-mtDNA or mtDNA copy number in neurological disorders.

Disease	Species	Sample	Finding	Ref.
Neurodegenerative diseases				
MS	*H. sapiens*	CSF samples of MS patients	Association between lower ccf-mtDNA and MS and neurodegeneration	[57]
MS	*H. sapiens*	MS patients’ blood plasma samples	Increased ccf-mtDNA in MS patients compared to healthy patients	[58,59]
MS	*H. sapiens*	MS patients’ CSF samples	Increased ccf-mtDNA in CSF of MS patients compared to cognitively healthy patients	[60]
MS	*H. sapiens*	Relapsing-remitting MS patients’ peripheral blood samples	Decreased ccf-mtDNA levels in MS patients’ when compared to healthy patients	[61]
PD	*H. sapiens*	CSF samples from PD patients	Reduced ccf-mtDNA in CSF from PD patients compared to healthy subjects	[62]
PD	*H. sapiens*	CSF samples from PD patients	Significant reduction in ccf-mtDNA in PD patients Reduction in ccf-mtDNA associated with type and duration of treatment	[63]
AD	*H. sapiens*	CSF samples from preclinical AD patients	Lower ccf-mtDNA levels in CSF of preclinical AD patients (several studies failed to replicate results)	[64]
ALS	*M. musculus*	In vivo study of aberrant TDP-43 transgenic mice	TDP-43 Caused mtDNA release to the cytoplasm via the mPTP	[29]
ALS	*H. Sapiens*	ALS spinal cord neuron cell line	ALS spinal cord neurons showed significant reductions in mtDNA-cn and mitochondrial gene deletions	[65]
**Neuropsychiatric diseases**				
Addiction	*H. sapiens*	Methamphetamine-addiction model in human dopaminergic neuroblastoma SH-SY5Y cell line	Decrease in mtDNA-cn Apoptosis after 48 h of treatment	[66]
Addiction	*M. musculus*	Peripheral blood from murine model of morphine addiction Peripheral blood from heroin addiction patients	Decrease in mtDNA-cn in hippocampi; Melatonin-dosage restored mtDNA-cn	[67]
Anxiety	*H. sapiens*	Serum samples from a middle-aged cohort	Increased ccf-mtDNA levels with introduction of negative mood stimulus	[68]
MDD	*H. sapiens*	Plasma samples from suicide attempters	Increased levels of ccf-mtDNA	[69]
MDD	*H. sapiens*	Plasma samples from unmedicated MDD patients	Elevated ccf-mtDNA levels compared to healthy subjects; No significant difference in peripheral blood mononuclear cells’ mtDNA-cn	[70]
SZ	*H. sapiens*	Patient blood plasma samples	Increase in ccf-mtDNA in SZ patients; mtDNA release because of exacerbated apoptosis	[71]
SZ BD	*H. sapiens*	Whole-blood samples of screened patients	Increasing age and psychosis severity correlated with decreasing ccf-mtDNA levels; Risperidone treatments found to have a reducing effect on ccf-mtDNA at concentrations simulating clinical target level in plasma, but not at concentrations simulating CSF or brain interstitial target level	[72]
BD type I (BD-I)	*H. sapiens*	BD-I patients’ leukocyte samples	Negative association between number of manic-episode relapses and mtDNA-cn	[73]
BD	*H. sapiens*	Serum samples from an adolescent cohort	No significant difference in ccf-mtDNA levels between groups; Increase in serum lactate levels for adolescents diagnosed with BD	[74]
BD	*H. sapiens*	Plasma samples from MDD patients	MDD patients showed lower plasma mtDNA levels than healthy patients	[75]
BD	*H. sapiens*	Serum samples from BD patients	Higher levels of ccf-mtDNA in BD patients	[76]

Table Abbreviations: major depressive disorder (MDD); bipolar disorder (BD); schizophrenia (SZ); multiple sclerosis (MS), Parkinson’s disease (PD), Alzheimer’s disease (AD); cerebrospinal fluid (CSF); mitochondrial DNA copy number (mtDNA-cn); circulating cell free mitochondrial DNA (ccf-mtDNA).

## Abbreviations

Adenine nucleotide translocase (ANT), adenosine triphosphate (ATP), Alzheimer disease (AD), amyloid-beta (Aβ), amyotrophic lateral sclerosis (ALS), bipolar disorder (BD), caspase 1 (CAS1), cerebrospinal fluid (CSF), circulating cell-free mitochondrial DNA (ccf-mtDNA), cyclophilin D (CyD), damage-associated molecular pattern (DAMP), double-stranded mitochondrial DNA (dsmtDNA), electron transport chain (ETC), fused in sarcoma (FUS), glutamate (Glu), GMP-AMP (cGAMP), GMP-AMP synthase (cGAS), hypothalamic-pituitary-adrenal (HPA), interferon (IFN), interferon regulatory factors (IRFs), interferon regulatory factor 3 (IRF3), interleukin (IL), knockout (KO), Leu-rich repeat kinase 2 (LRRK2), major depressive disorder (MDD), methionine (Met), methylenetetrahydrofolate dehydrogenase (NADP^+^-dependent) 1-like protein (MTHFD1L), mitochondrial cytochrome c oxidase I (MTCO1), mitochondrial DNA (mtDNA), mitochondrial NADH dehydrogenase III (MTND3), mitochondrial permeability transition pore (mPTP), mitochondrial reactive oxygen species (mtROS), mitochondrial ubiquinol:cytochrome c reductase complex cytochrome b subunit (MTCYB), multiple sclerosis (MS), NOD-like receptor, pyrin containing protein 3 (NLRP3), nuclear factor-kB (NF-κB), optineurin (OPTN), oxidative phosphorylation (OXPHOS), Parkinson disease (PD), pro-caspase 1 (pro-CAS1), pro-interleukin (pro-IL), reactive oxygen species (ROS), schizophrenia (SZ), single-stranded mitochondrial DNA (ssmtDNA), superoxide dismutase-1 (SOD1), superoxide dismutase-2 (SOD2), TANK-binding kinase 1 (TBK1), TAR DNA-binding protein (TDP-43), Toll-like receptor 9 (TLR9), tumor necrosis factor-ɑ (TNF-ɑ), voltage-dependent anion channel (VDAC).
